# Taste-Masked Flucloxacillin Powder Part 1: Optimisation of Fabrication Process Using a Mixture Design Approach

**DOI:** 10.3390/ph16081171

**Published:** 2023-08-17

**Authors:** Okhee Yoo, Sam Salman, Britta S. von Ungern-Sternberg, Lee Yong Lim

**Affiliations:** 1 Pharmacy, School of Allied Health, University of Western Australia, Perth, WA 6009, Australia; okhee.yoo@uwa.edu.au; 2 Clinical Pharmacology and Toxicology Unit, PathWest Laboratory Medicine, Perth, WA 6009, Australia; sam.salman@uwa.edu.au; 3 Medical School, The University of Western Australia, Perth, WA 6009, Australia; britta.regli-vonungern@uwa.edu.au; 4 Perioperative Medicine Team, Perioperative Care Program, Telethon Kids Institute, Perth, WA 6009, Australia; 5 Department of Anaesthesia and Pain Medicine, Perth Children’s Hospital, Perth, WA 6009, Australia

**Keywords:** flucloxacillin, Eudragit EPO, palmitic acid, taste-masked microparticles, mixture design, paediatric formulation

## Abstract

It is extremely challenging to formulate age-appropriate flucloxacillin medicines for young children, because flucloxacillin sodium (FS) has a lingering, highly bitter taste, dissolves quickly in saliva, and requires multiple daily dosing at relatively large doses for treating skin infections. In this paper, we describe a promising taste-masked flucloxacillin ternary microparticle (FTM) formulation comprising FS, Eudragit EPO (EE), and palmitic acid (PA). To preserve the stability of the thermolabile and readily hydrolysed flucloxacillin, the fabrication process employed a non-aqueous solvent evaporation method at ambient temperature. Optimisation of the fabrication method using a mixture design approach resulted in a robust technique that generated stable and reproducible FTM products. The optimised method utilised only a single solvent evaporation step and minimal amounts of ICH class III solvents. It involved mixing two solution phases—FS dissolved in ethanol:acetone (1:4 *v*/*v*), and a combination of EE and PA dissolved in 100% ethanol—to give a ternary FS:EE:PA system in ethanol: acetone (3:1 *v*/*v*). Solvent evaporation yielded the FTMs containing an equimolar ratio of FS:EE:PA (1:0.8:0.6 *w*/*w*). The fabrication process, after optimisation, demonstrated robustness, reproducibility, and potential scalability.

## 1. Introduction

**Figure 1:**
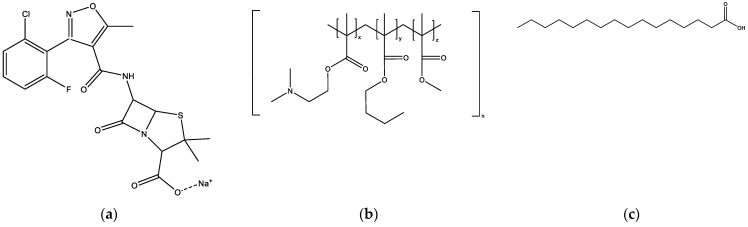
Chemical structures of (**a**) flucloxacillin sodium; (**b**) Eudragit EPO, which consists of dimethylaminoethyl methacrylate (x), butyl methacrylate (y), and methyl methacrylate (z), at a ratio of 2:1:1; (**c**) palmitic acid.

Flucloxacillin is an antibiotic with a lingering, highly bitter taste. It is available commercially in oral liquid formulations to treat staphylococcal skin and soft-tissue infections in young children. Commercial liquid flucloxacillin medicines are very poorly accepted because of their foul taste [1], making it extremely challenging to achieve compliance for dosing four times a day in children for effective therapeutic management. Flucloxacillin is administered at relatively high doses, with children over one month of age requiring 12.5 to 25 mg/kg (maximum of 1 g) every 6 h [2]. This imposes a demand for a high drug-to-matrix ratio for flucloxacillin medicinal products. Flucloxacillin is a strong acid (pKa 2.76) and is supplied commercially as flucloxacillin sodium (Figure 1, FS), which is hygroscopic and highly soluble (1 g/mL) [3] in aqueous media, including saliva [4]. Also, FS has a fused thiazolidine ring and a highly strained β-lactam ring that makes it thermolabile and prone to hydrolysis [5]. These FS properties make it highly challenging to develop a child-appropriate taste-masked FS medicinal product.

There is limited published literature on the development of solid oral age-appropriate medicines for young children that successfully mask the taste of highly bitter drugs that are also very water-soluble and require high drug loading (>50 mg) per dosage unit. A common technique is to incorporate the drug into a fine particle matrix using a range of manufacturing techniques [6,7,8]. The resultant microparticles are administered as such, or they may be further processed into chewable and orally dispersible tablets to facilitate accurate dosing to young children. To reduce the perception of grittiness and ease swallowability, finer particles (threshold = 300 µm) are desirable [9]. Conversely, particles of 500–710 µm are reported to be gritty [10], although the perception of grittiness is also dependent on the excipients [9], amount of particles, and viscosity of the vehicle administered [10]. For FS, formulation into very fine particles may not be practical, as the limited capacity of small particles to achieve high drug loading and restrict drug leakage in the oral cavity may necessitate a high particle load per dose that adversely affects palatability and safety.

Of the polymers employed for particulate formulations, Eudragit EPO (Figure 1, EE) has a solubility profile that is favourable for masking the taste of bitter drugs. EE is a cationic copolymer consisting of dimethylaminoethyl methacrylate, butyl methacrylate, and methyl methacrylate (molar ratio 2:1:1) [11] that dissolves at acidic pH values of less than 5. This pH-dependent solubility may enable an EE coating to minimise the interaction of a drug load with taste receptors without affecting the drug’s bioavailability in the lower GIT. EE, being cationic, also reacts with acidic drugs to form a reversible complex [12], which may provide another mechanism for masking the taste of flucloxacillin [13]. Additionally, as FS is sensitive to heat and moisture levels above 1% [14], a fabrication process that avoids water and heating is desired, and EE is soluble in ethanol and acetone, which are both class III solvents in the ICH Q3C (R5) guidelines with low boiling points [15]. Class III solvents have low toxicity to humans (daily permitted dose of 50 mg or more) and are the preferred solvents in pharmaceutical processing.

EE was found in our preliminary experiments to form a solid solution with FS, and the solid solution was transformable to a fine powder formulation through the addition of palmitic acid (Figure 1, PA) as a fatty acid co-carrier. Complexation of EE with a fatty acid has been applied to improve the taste of drug formulations [16,17]. Relatively polar lipids like PA are preferred, as the nonpolar lipids (e.g., triglycerides) cause phase separation due to inadequate miscibility with the FS-EE solid solution. Additionally, PA has a relatively low melting point (63 °C), which is advantageous for processing with the thermolabile FS, and PA is cheaper to purchase than the more novel polar lipids (e.g., Precirol ATO 5™). PA has also been used for masking the taste of the bitter antibiotic cefuroxime axetil [18].

The aim of this study was to apply a design-of-experiments approach to optimise a fabrication process for a flucloxacillin ternary microparticle (FTM) formulation using FS, EE, and PA. In preliminary experiments, we were able to generate FS-EE-PA microparticles by two methods: In the first method, FS and EE were levigated in ethanol, followed by solvent evaporation of the suspension over 30 min at ambient temperature to obtain an FS-EE solid solution. The solid solution was redissolved in acetone, added dropwise to molten PA, and evaporated to dryness at ambient temperature over 24 h to obtain a solid, which was then ground into microparticles. The second method bypassed the formation of the FS-EE solid solution and the melting of PA. Instead, FS, EE, and PA were co-triturated in acetone or ethanol, and the suspension was transformed to a solid by solvent evaporation over 24 h at ambient temperature. Both methods yielded comparable powders; however, the second method was favoured due to the smaller number of processing steps, the avoidance of heat processing, and its robustness under different environmental conditions. However, the FS-EE-PA powders in the second method were prepared using a suspension of FS, EE, and PA particles. Solutions are preferred over suspensions, as a suspension contains particles of different sizes and does not provide optimal conditions for molecular interactions between the components to give greater assurance of a homogeneous and reproducible FTM product. This paper describes the design-of-experiments approach undertaken to develop a robust technique whereby FS, EE, and PA were all solubilised using minimal amounts of class III solvents in the ICH Q3C (R5) guidelines to consistently yield a stable and taste-masked FS-EE-PA microparticle formulation.

## 2. Results

### 2.1. Optimisation of the Fabrication Process

The single ternary-phase system, comprising 0.2 g of FS, 0.16 g of EE, and 0.12 g of PA, could only be co-solubilised with ethanol alone, using 25 mL of ethanol. The other four acetone-containing solvent systems were not able to achieve co-solubilisation of the three components when applied at up to 25 mL.

**Figure 2:**
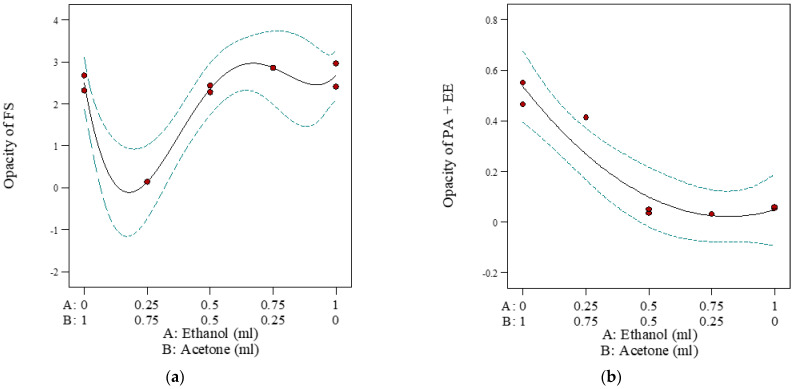
Predicted model graph to determine optimal solvents for the two-phase systems in Option 1: (**a**) Predicted model graph of phase 1 showing opacity measured as a function of the proportion of ethanol to acetone. (**b**) Predicted model graph of phase 2 showing opacity measured as a function of the proportion of ethanol to acetone. A: proportion of ethanol; B: proportion of acetone volume. The blue line denotes the 95% confidence bands, while the red dots indicate design points.

For the two-phase systems, only in Option 1 were both the binary and single-component phases able to be solubilised with the solvents employed. The optimal proportion of ethanol to acetone required to solubilise each phase was predicted by a minimised opacity, measured as the absorbance at 550 nm. The results are shown in Table 1, and the summary statistics of the predicted model coefficients are shown in Table 2. Both models for phase 1 and phase 2 were significant, with *p*-values of 0.02 and 0.025, respectively. However, the lack of fit for phase 2 was significant, and the lack of fit for phase 1 was not predicted. This was partly due to the very small error of the replicas, which does not imply a lack of model fit, as can be seen in Figure 2. The r^2^ statistics of the models for phases 1 and 2 were significant, at 0.9593 and 0.9089, respectively. The insignificant coefficient (AB) of the model, where the 95% CI contained 0, was included when the term was required to support the hierarchy of parameters in the model. The term with the larger coefficient was the more influential variable on the response (i.e., solubility) than the term with the smaller coefficient. As shown in Table 2, ethanol (coefficient 2.69) and acetone (coefficient 2.50) equally influenced the solubility of FS (phase 1), and the interaction terms (AB(A-B), coefficient 13.99, and AB(A-B)2, coefficient −19.52) had a higher influence on solubility than the individual solvents. The optimal proportion of ethanol to acetone required to solubilise FS (phase 1) was determined to be 1:4, at the lowest point of the opacity model graph (Figure 2a). With the EE-PA binary phase (phase 2) of Option 1, transparent solutions were observed when 2 mL of any of the solvents was added. The visual observations were supported by low levels of measured opacity (consistently below 0.55, Table 1) for all samples, and smaller values for the model coefficients (Table 2). The collective data suggest that the proportion of ethanol to acetone did not significantly influence the co-solubilisation of PA and EE. However, there was a trend towards lower opacity at higher ethanol content (Figure 2b); hence, ethanol alone was regarded as the optimal solvent for phase 2.

**Figure 3:**
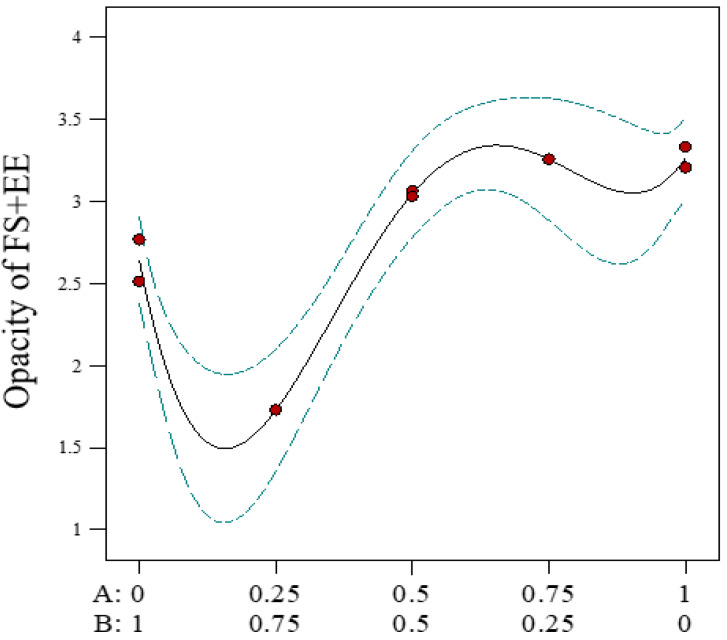
Predicted model graph to determine optimal solvents for phase 2 (FS-EE) of Option 3 of the two-phase systems, showing opacity measured as a function of the proportion of ethanol to acetone. A: proportion of ethanol; B: proportion of acetone volume. FS = flucloxacillin sodium, EE = Eudragit EPO. The blue line denotes the 95% confidence bands, while the red dots indicate design points.

For Option 2 of the two-phase systems, phase 1 (EE) was soluble in 1 mL of all specified solvents; however, phase 2 (FS and PA) could not be dissolved in 2 mL of any of the solvents. For Option 3, phase 1 (PA) was insoluble in 1 mL of all specified solvents, while phase 2 (FS and EE) showed a similar opacity model graph to that for phase 1 (FS) of Option 1, with minimal opacity predicted at an ethanol volume ratio of approximately 0.2 (Figure 3). These results suggest that EE did not influence the solubilisation of FS in the specified solvents, whereas EE did influence the solubilisation of PA.

**Figure 4:**
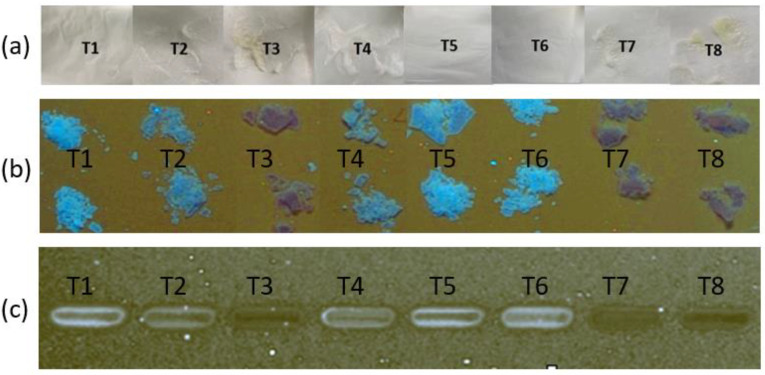
Images of powders prepared by drying (at ambient temperature for 48 h) ternary suspensions prepared by triturating 0.2 g of FS, 0.16 g of EE, and 0.12 g of PA with 3 mL of solvent. Images were obtained of powder samples with (**a**) a camera, and with high-performance thin-layer chromatography (**b**) before and (**c**) after solubilisation in ethanol. FS = flucloxacillin sodium, EE = Eudragit EPO, PA = palmitic acid.

**Figure 5:**
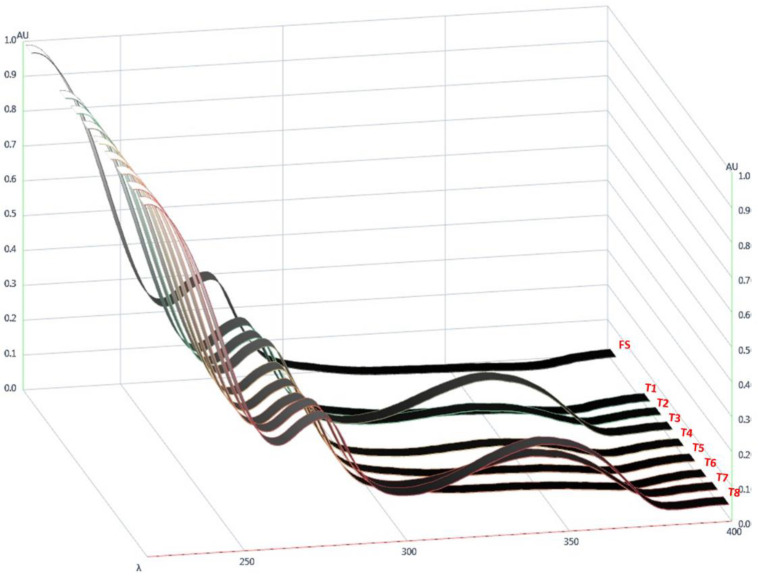
UV spectra of flucloxacillin sodium (FS) and powders obtained by drying ternary systems of flucloxacillin sodium, Eudragit EPO, and palmitic acid prepared using different solvents (T1–T8).

The next stage of the optimisation study was to determine the optimal solvent ratio that would generate stable FTMs from non-solubilised ternary systems. Discolouration of samples was examined as a surrogate measure of drug stability, as FS turned yellow on degradation, which was confirmed by HPLC analysis. Visual examination indicated that T3 and T8, both prepared with acetone alone as the solvent, had the strongest yellow colour, followed by T7, prepared with 0.25:0.75 *v*/*v* of ethanol:acetone, while T2 and T4, both prepared with 0.5:0.5 *v*/*v* of ethanol:acetone, were slightly yellow (Figure 4a). Thus, samples prepared with solvents containing a higher proportion of acetone were associated with more intense yellow colouration. The differential discolouration was amplified when images of the samples were taken using HPTLC and processed by ImageJ, with the powder samples before (Figure 4b) and after solubilisation with ethanol (Figure 4c) showing similar discolouration trends to the visual observations. In addition, the diode-array UV spectra of the samples indicated that the height of the absorption peak at 343 nm was related to the yellow discolouration, with greater absorption shown by the more intensely yellow samples (Figure 5).

**Figure 6:**
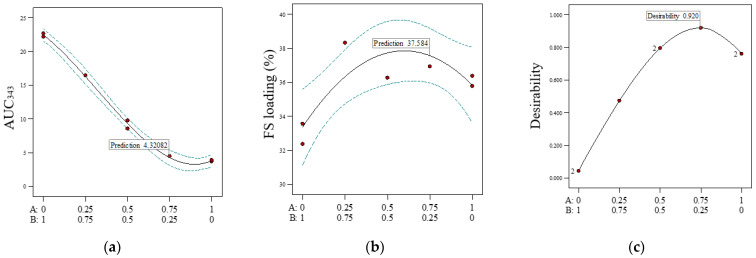
Predicted model graphs of (**a**) AUC_343_, (**b**) FS loading efficiency (%), and (**c**) desirability, where AUC_343_ is minimised and FS loading efficiency (%) is maximised, as a function of the proportion of ethanol in the solvent system used to prepare the ternary samples of flucloxacillin sodium (FS), Eudragit EPO, and palmitic acid. A: proportion of ethanol; B: proportion of acetone volume. The blue line denotes the 95% confidence bands, while the red dots indicate design points.

As the yellow discolouration of samples was correlated with a higher UV absorbance at 343 nm, the UV absorption at 343 nm was quantified using the validated HPLC assay developed for FS. The AUC_343_, measured as the sum of all HPLC peaks detected at 343 nm, is shown in Table 3. AUC_343_ was strongly correlated with the proportion of acetone used to prepare the samples (Figure 6a). The FS loading efficiency in the samples was determined using the same HPLC method, with the eluent monitored at 225 nm. Both models were found to be significant, while the lack of fit for both was determined to be non-significant, as detailed in Table 4. The r^2^ values for the models of drug-loading efficiency and AUC_343_ were 0.7358 and 0.9978, respectively. The statistically significant model parameters (*p* < 0.05) and CI (Table 4) suggested that ethanol and acetone equally influenced the drug-loading efficiency (A, coefficient 35.84, and B, coefficient 33.38; Table 4), while acetone was the stronger influencing factor for AUC_343_ (A, coefficient 3.82, and B, coefficient 22.47; Table 4).

**Figure 7:**
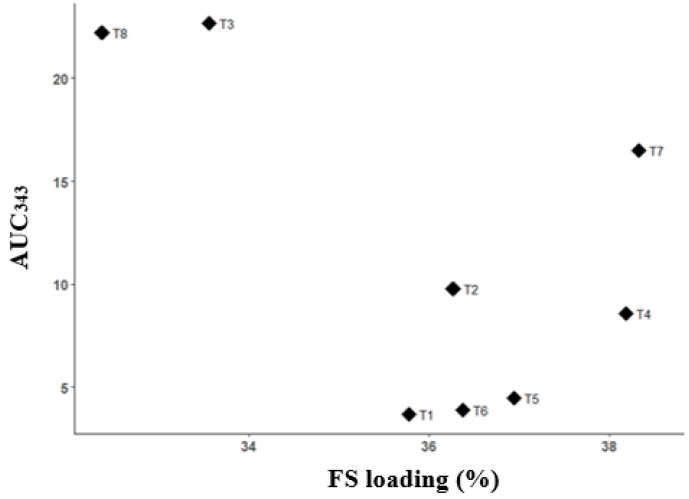
Scatterplot of FS loading efficiency (%) vs. AUC_343_ for powders obtained by drying ternary systems of flucloxacillin sodium (FS), Eudragit EPO, and palmitic acid prepared using different solvents (T1–T8).

The goal of the model prediction was to find the volume ratio of ethanol to acetone that minimised AUC_343_ and maximised the FS loading efficiency. A plot of FS loading efficiency versus AUC_343_ showed samples T3 and T8 to occupy the top-left corner, i.e., having very low FS loading efficiency and very high AUC_343_ (Figure 7). There appeared to be an inverse relationship between the drug-loading efficiency and the AUC_343_ (Figure 7), with the lowest drug-loading efficiency and highest AUC_343_ observed at 100% acetone. However, the relationship between the drug-loading efficiency and the AUC_343_ was less sensitive at drug-loading efficiency over 36%, where drug degradation was at minimal levels that were not significantly different from experimental noise.

To consider both responses (drug-loading efficiency and AUC_343_) simultaneously, a new response called the desirability index was defined. Each response was ranked within its own span in the design space, and the geometric mean of the ranked responses was defined as the desirability index. The desirability index ranged from 0 to 1, and a new model was predicted based on the desirability index against the volume proportion of ethanol. The optimal volume proportion of ethanol to acetone, defined as that which yielded the highest desirability index, was identified from the model to be 75:25 (Figure 6c). This prediction was in agreement with the experimental data, where the maximum acetone content for generating FTMs without a visible yellow discolouration was 25% *v*/*v* (T5 sample).

The final solvent system for the formulation of the FTMs was based on the optimal solvent systems predicted for the Option 1 two-phase systems and the ternary-phase systems. In Option 1, the optimal solvent predicted to solubilise phase 1 (FS) was 20% ethanol and 80% acetone, while the optimal solvent predicted to solubilise phase 2 (EE + PA) was 100% ethanol. When these two phases were combined, the final ternary phase should contain 75% ethanol and 25% acetone based on the optimal solvent system predicted to yield a stable FTM product. To achieve these goals, the volume of ethanol required to solubilise phase 2 was calculated as follows: Firstly, the minimum volume of the optimal solvent (20% ethanol and 80% acetone) required to dissolve FS (phase 1) was determined, and this was 3.68 mL/g (2.88 *g*/*g*). This volume was divided into the volumes of ethanol and acetone for phase 1, based on the optimal ratio of 20:80. The calculated volume of acetone would represent the acetone present in the ternary mixture, since phase 2 did not require acetone for solubilisation. Thus, the amount of acetone was predetermined for the final ternary mixture, and from this, the corresponding volume of ethanol required for the final ternary mixture was calculated based on the optimal ethanol-to-acetone ratio of 75:25 (*v*/*v*). Finally, by subtracting the volume of ethanol required in phase 1 from the volume of ethanol required for the final ternary mixture, the volume of ethanol required for phase 2 was determined. An example of this calculation to prepare FTMs using 1 g of FS, 0.8 g of EE, and 0.6 g of PA is shown in Table 5.

### 2.2. Confirmation of the Optimal Fabrication Process

**Figure 8:**
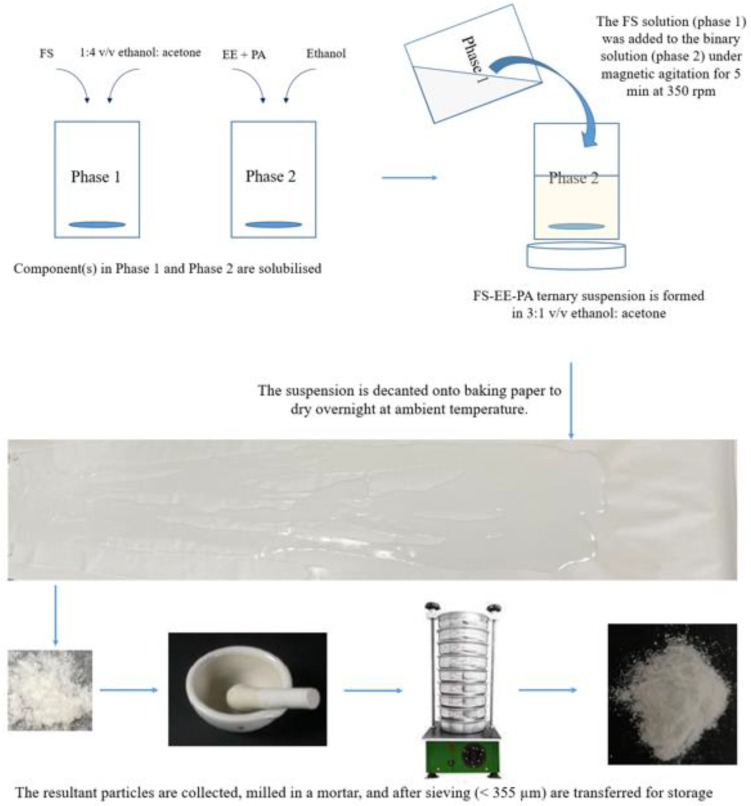
Schematic diagram showing the optimised fabrication process for preparing a taste-masked powder from a ternary system comprising flucloxacillin sodium (FS), Eudragit EPO (EE), and palmitic acid (PA).

**Figure 9:**
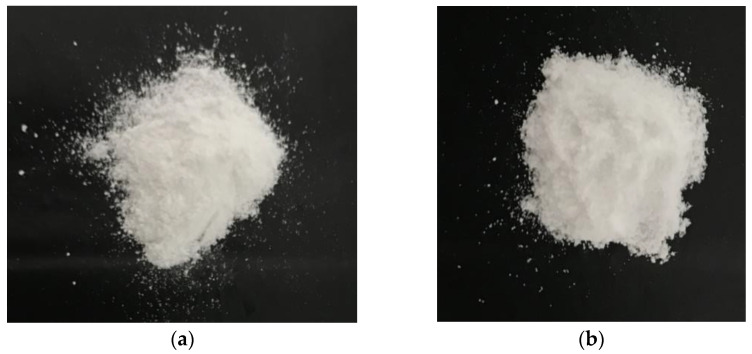
FTM images: (**a**) produced via the optimised fabrication method at baseline; (**b**) after 6 months of storage at ambient temperature.

**Figure 10:**
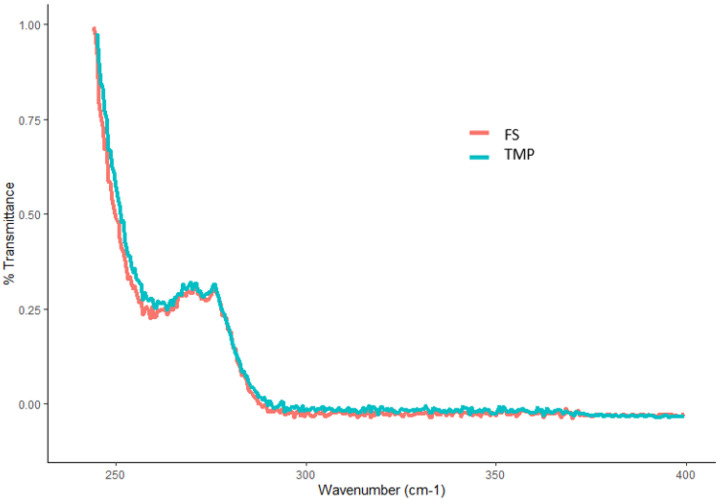
UV spectra of an FTM sample produced by the optimal fabrication process (light blue) and flucloxacillin sodium (FS, pink).

A schematic diagram showing the optimal fabrication process for the final FTMs is depicted in Figure 8. Six independent batches of FTMs were prepared using this process: three within the same day (intraday), and another three batches on another day (interday). White powders were obtained for all batches, and their colour and texture were consistent across the batches (Figure 9). The UV spectra for the batches did not show any peak at 343 nm, and all were almost identical to the UV spectrum for the FS control sample; a typical overlay is shown in Figure 10. The drug-loading efficiency was high and reproducible, with the mean drug-loading efficiency in each of the six batches within a narrow range of 39.1 to 40.8% *w*/*w* (Table 6), against a predicted drug-loading efficiency of 41.7% *w*/*w*. There was also uniformity of drug-loading efficiency within batches (SD < 1%).

### 2.3. Differential Scanning Calorimetry (DSC)

**Figure 11:**
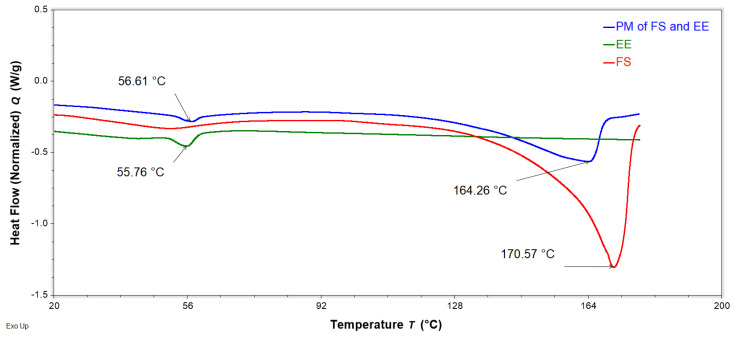
DSC thermograms of neat flucloxacillin sodium (FS, red), neat Eudragit EPO (EE, green), and a binary physical mixture (PM) of FS and EE (1:0.8 *w*/*w*, blue) obtained with a heating rate of 10 °C/min, showing the peak temperature of the endotherms of FS, EE, and PM.

**Figure 12:**
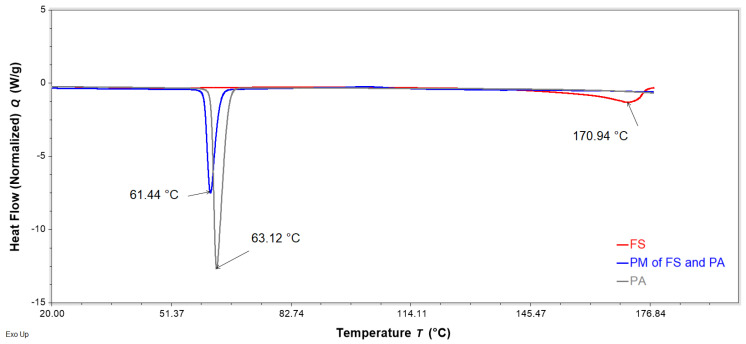
DSC thermograms of neat flucloxacillin sodium (FS, red), neat palmitic acid (PA, grey), and a binary physical mixture (PM) of FS and PA (1:0.6 *w*/*w*, blue) obtained with a heating rate of 10 °C/min, showing the peak temperature of the endotherms of FS, PA, and PM.

**Figure 13:**
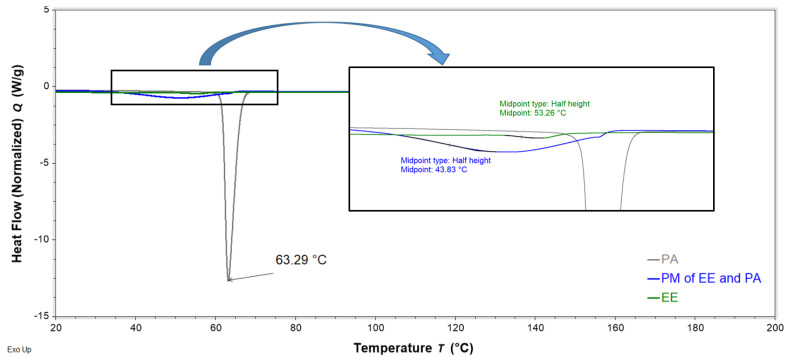
DSC thermograms of neat Eudragit EPO (EE, green), neat palmitic acid (PA, grey), and a binary physical mixture (PM) of EE and PA (0.8:0.6 *w*/*w*, blue) obtained with a heating rate of 10 °C/min, showing the glass transition temperatures of EE and PM, as well as the peak temperature of the endotherm of PA. Phase transitions between 30 and 70 °C are amplified in the zoom box.

**Figure 14:**
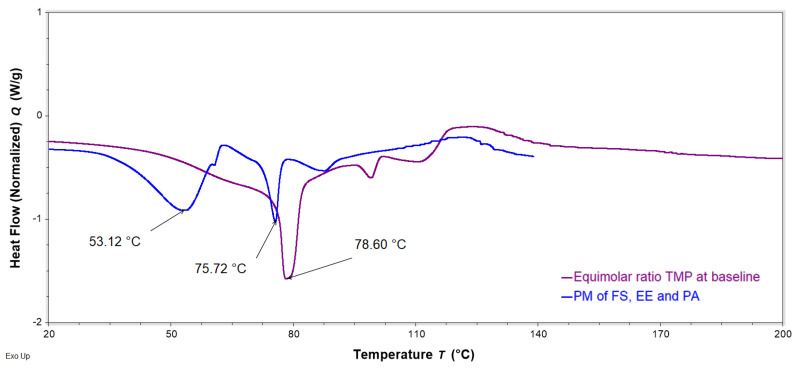
DSC thermograms of FTMs (purple) fabricated using the optimised method and the ternary physical mixture (PM) (FS:EE:PA at 1:0.8:0.6 *w*/*w*/*w*, blue) obtained with a heating rate of 10 °C/min, showing the peak temperature of the endotherms of FTMs and PM. The analysis was not prolonged, due to the material degradation associated with temperature above 100 °C. FS = flucloxacillin sodium, EE = Eudragit EPO, PA = palmitic acid.

**Figure 15:**
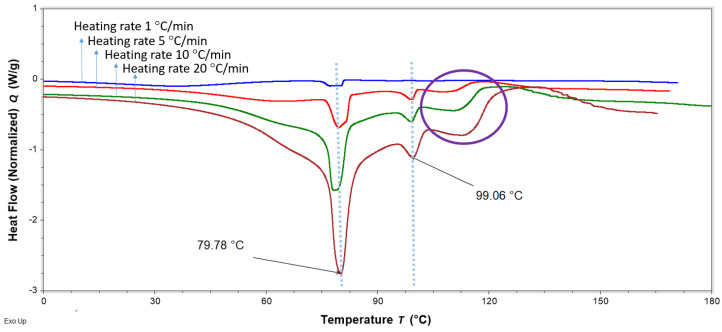
DSC thermograms of FTMs fabricated using the optimised method (FS:EE:PA at 1:0.8:0.6 *w*/*w*/*w*), heated at different heating rates of 1 to 20 °C/min, showing variations in the phase transitions above 100 °C. The likely degradation events are circled in purple. FS = flucloxacillin sodium, EE = Eudragit EPO, PA = palmitic acid.

DSC analysis was performed to evaluate interactions of FS, EE, and PA in the FTMs. FS and PA are crystalline solids with reported melting temperatures of 176–178 °C [19] and 63 °C [20], respectively, whereas the co-polymeric EE is amorphous, with a reported Tg of 57 °C [21]. PMs were prepared by triturating the specified component powders for 3 min using a pestle and mortar. DSC thermograms of the binary PM of FS and EE (1:0.8 *w*/*w*) showed the retention of the melting endotherm of FS (approximately 170 °C) and the glass transition of EE (56 °C), suggesting a lack of interaction between these two components (Figure 11). Conversely, the DSC thermogram of the PM of FS and PA (1:0.6 *w*/*w*) exhibited only a single endotherm, with a peak temperature (61 °C) slightly lower than that of the melting endotherm of neat PA (63 °C) (Figure 12). The absence of the FS melting endotherm suggests the transformation of the drug from crystalline to amorphous form. The DSC thermogram for the PM of EE and PA (0.8:0.6 *w*/*w*) did not exhibit the PA melting endotherm, and a glass transition lower than that of EE was observed at around 44 °C (Figure 13). This suggests a transition of PA from crystalline to amorphous phase, potentially molecularly distributed in the EE phase. The DSC thermogram of the ternary PM (FS:EE:PA at 1:0.8:0.6 *w*/*w*/*w*) showed a glass transition at 53 °C and an endotherm at 76 °C, which was not observed in the DSC thermograms of the binary PM samples (Figure 14). By comparison, the FTMs fabricated using the optimised method did not show the glass transition, but it had a prominent broad endotherm with a peak temperature of 78.6 °C (Figure 14). DSC analyses of the FTMs at different heating rates of 1 to 20 °C/min showed variations in the phase transitions above 100 °C (Figure 15), suggesting that these were likely to be associated with material degradation. On the other hand, the endotherms with peak temperatures at around 80 °C and 99 °C did not change significantly with the heating rate, and they might be considered to be true melting endotherms of the FTMs [22].

## 3. Discussion

Using the Option 1 two-phase systems, a total of 3 mL of solvent was adequate to solubilise all three components, which was considerably smaller than the 25 mL of ethanol required to solubilise the single ternary-phase system. When the solubilised phase 1 and phase 2 were combined, the ternary system was a suspension, which could be dried on baking paper to yield the FTMs. Using smaller volumes of solvent to generate the FTMs is advantageous, as it is more cost-effective, the higher sample load per unit volume of solvent requires less solvent and a shorter drying time, and it improves handling. The evaporation of a larger amount of organic solvent is also not environmentally friendly, and the longer processing time may lead to greater variation in the final product quality.

Visual inspection provides a quick and popular method to evaluate components’ solubilisation in a solvent [12], and this was employed to ascertain the co-solubilisation of FS, EE, and PA in the single ternary system, where increasing amounts of solvent were added to achieve solubilisation. Conversely, in the evaluation of the two-phase systems, it was desired to employ a minimal amount of solvent to dissolve the phases, and there were a potentially infinite number of solvent combinations that could be employed to identify the optimal solvent combination. Using a modelling approach, the optimal solvent ratio was able to be predicted from experiments using a limited number of solvent combinations. To use the mixture design methodology to predict the optimal solvent for each phase, a quantitative measure of turbidity [23] had to be adopted as a model response. PA alone was found not to be soluble in any combination of ethanol and acetone at the specified volumes of solvent used; however, PA could be solubilised in the solvents in the presence of an equimolar amount of EE. This could be attributable to the formation of an EE palmitate salt (Equation (1)), as EE is known to form an aqueous soluble salt with stearic acid [24], which is a similar saturated fatty acid but with a longer carbon chain (C18) compared to palmitic acid (C16). FS could also interact with EE (Equation (3)) and PA (Equation (2)); however, the solubility of FS was not affected by either EE or PA. The solubilisation of FS was not achievable with ethanol alone; it required the presence of acetone at the volume of solvent employed.

The optimal solvent ratio for the ternary phase was determined to maximise the drug-loading efficiency and minimise the AUC_343_ for the FTMs. An inverse relationship between the drug-loading efficiency and the AUC_343_ was noted, and it was clear that higher AUC_343_ was correlated with a higher acetone ratio used to prepare the FTMs. Residual acetone in the FTMs could not have accounted for the measured AUC_343_, as acetone absorbs in the UV range of 195 to 275 nm [25] and has an absorbance cutoff of 330 nm [26]. Flucloxacillin is a penicillin analogue. The main degradation product of penicillin is penicillenic acid, which also is known to cause allergy [14]. Penicillenic acid absorbs strongly at 322 nm [14], and as its side chain changes, the absorption peak can shift. Thus, AUC_343_, a measure of the absorbance at 343 nm, could be attributed to a penicillenic acid analogue—the likely cause of the yellow discolouration of flucloxacillin. The confirmation study showed that this yellow discolouration occurred only in ternary systems in which the solvent had an acetone content higher than 25%. FS alone did not show discolouration when dissolved in a solvent with a high acetone content of 80%. Thus, the presence of EE and/or PA in a solvent containing acetone (>25%) might have facilitated the degradation of FS.

The optimised fabrication process for the FTMs using the two-phase system was designed to satisfy two conditions: Firstly, the FS-alone phase had to be solubilised in its optimal solvent (ethanol to acetone = 20:80 *v*/*v*). Secondly, the solvent in the final ternary phase had to contain less than 25% acetone. The twin conditions were achieved by increasing the volume of ethanol used to solubilise the binary EE-PA phase. The two-phase fabrication process yielded reproducible FTM products of reproducible colour, texture, AUC_343_ profile, and drug-loading efficiency.

The DSC analysis suggested interaction between PA and FS in the binary PM. The presence of FS depressed the melting temperature of PA due to the ‘impurity’ effect. During the DSC scan, the FS crystals appeared to have dissolved in the molten PA, as the FS melting endotherm was no longer detectable in the thermogram for the PM. FS is a conjugated salt of an acid (flucloxacillin, pKa 2.76 [4]) and is able to abduct hydrogen from the molten PA (weak acid, pKa 4.75 [20]) to form sodium palmitate, akin to the formation of an acid–soap by a fatty acid in an alkaline buffer [27]. Similarly, the disappearance of the PA melting peak in the DSC thermogram of the binary PM of PA and EE might be attributed to the crystalline PA having been solubilised via acid–base interactions with EE above the Tg of the polymer. The DSC data therefore support the solubilisation data, where PA was observed to be more readily solubilised in the presence of EE than alone in the solvent systems tested. On the other hand, EE and FS were not found to interact when co-analysed in the DSC.

DSC analysis of the FTMs did not show the transitions attributable to the individual components; instead, there was a strong endotherm at 78.6 °C and a smaller endotherm at 99 °C, both of which were not observed with the binary PM samples. This suggests the formation of a new material that might result from specific intermolecular interactions when solutions of FS, EE, and PA are combined. The proposed reaction mechanisms can be summarised as follows: (1) PA and EE become ionised when co-solubilised in ethanol, and PA donates a hydrogen from its carboxylic acid group to the tertiary amine groups of EE; (2) when the solution of PA and EE is mixed with the FS solution, the sodium counterion of FS is transferred to PA to form a salt; and (3) the ionised flucloxacillin forms ionic bonds with the positively charged EE. The immediate precipitation of PEC occurs at steps (2) and (3), and to form this reaction the drug has to be present as the sodium salt.

Control experiments performed with the ternary PM also produced an endotherm at 75.7 °C after the glass transition of EE. Thus, it appeared that a similar material to that in the FTMs was generated during the DSC scan of the ternary PM. The ternary PM consisted of a mixture of FS, EE, and PA powders triturated together, and it would not be surprising for the molecular interactions to occur only after the EE transitioned from a glassy state to a rubbery state, when it was able to solubilise the PA to initiate the chain of reactions. The DSC data also suggest that FS interacted with EE only in the presence of PA, which further confirms the requirement of sodium ions from FS.

The FTMs are therefore a polyelectrolyte complex (PEC) with EE as the polycation and FS and PA as anions. PECs where polycations or polyanions spontaneously pair up with their counterions to form thermodynamically stable bonds [28] have been utilised to mask the taste of bitter drugs [29]. In general, PECs are prepared by separately dissolving the two oppositely charged components in suitable solvents and then mixing the two solutions and collecting the precipitated PEC by centrifugation and washing to prevent excess components from adhering to the formed PEC [30]. In this study, the final mixture obtained from mixing the FS solution with the EE + PA binary solution was a milk-like suspension, i.e., coacervates were formed. EE is considered to be a weak polyelectrolyte, as its ionisation status is dependent on pH [31], and it appears that the ionic strength for the generation of the FTMs was not strong enough to form solid precipitates [32]. Unlike most published methods, post-manufacture centrifugation was not applied to avoid the formation of aggregates. Post-manufacture washing was also not employed, so as to minimise drug loss, as FS is a highly water-soluble drug that has the potential to leach readily into the wash, resulting in low drug-loading efficiency for the FTMs. Instead, the entire ternary suspension was decanted onto baking paper for solvent evaporation to isolate the FTMs. With this method of drying, there is the risk of unbound FS giving the FTMs a bitter taste, and the unbound PA or EE can have the potential to affect drug dissolution rates. The risk of physisorption of excess unbound components can be minimised by preparing the optimal component ratio of FS, EE, and PA based on the reaction stoichiometry, and by using this to prepare the FTMs. A second study to investigate the optimal component ratio that would provide the optimal taste masking and dissolution profile was undertaken and is described in a separate manuscript.

Another limitation of the drying process relates to scaled-up manufacture. Although consistent batches of FTMs were prepared at the laboratory scale, the drying method may not be practical for large-scale manufacturing, as it requires a large drying surface to ensure consistency of the product. An inadequate drying surface leads to product pileup that traps too much solvent and slows down the drying time [33]. In addition, the concentration gradient that is created within the film as the solvent from the surface evaporates can generate compositional gradients [34] that lead to non-uniformity of drug distribution in the FTMs. Indeed, the FTMs prepared from thinner drying films have been found to contain feather-like particles that were not present in FTMs obtained from thicker drying films. To circumvent this, an automated spreading of the ternary mixture onto a drying tray to form drying films of consistent thickness, coupled with the provision of cold gentle air for instantaneous drying of the film at low temperatures, may be appropriate for large-scale production of the FTMs.

Finally, the taste-masking of FTMs has not been assessed by an independent taste panel, which would require further study.

## 4. Materials and Methods

### 4.1. Materials

Flucloxacillin sodium (FS) monohydrate was purchased from Aspen Pharmacare Australia Pty Ltd. (St Leonards, NSW, Australia). Eudragit^®^ EPO was gifted by Evonik (Evonik Industries, Essen, Germany). Palmitic acid was purchased from Acros Organics (Morris Plains, NJ, USA), ethanol was purchased from Chem-Supply Pty Ltd. (Gillman, SA, Australia), and acetone was purchased from RCI Labscan (Bangkok, Thailand). Potassium dihydrogen phosphate was purchased from Unilab (Sydney, NSW, Australia). Unless specified, all chemicals and reagents were of analytical grade. This study utilised five solvent systems comprising ethanol:acetone at the following volume ratios: 1:0, 0.75:0.25, 0.5:0.5, 0.25:0.75, and 0:1.

### 4.2. Methods

#### 4.2.1. Preliminary Study

**Figure 16:**
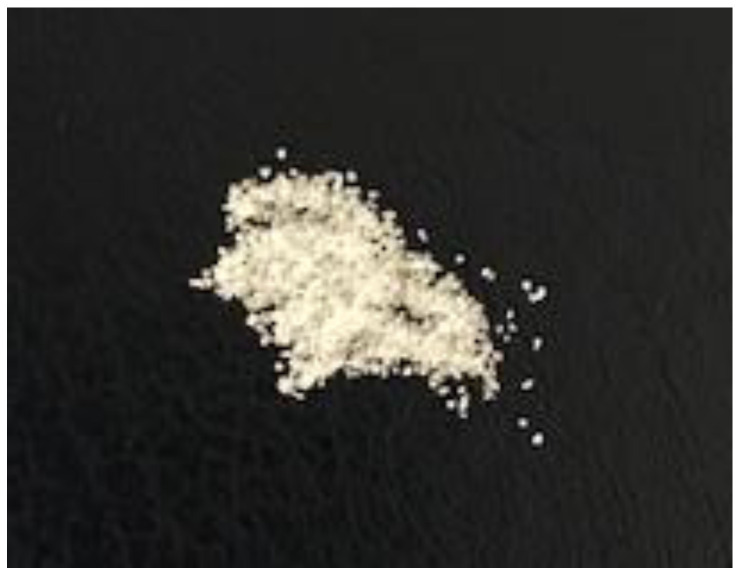
FS-EE-PA microparticles that passed through a sieve with a pore diameter of 355 µm and were retained on sieves with pore diameters of 212 µm.

The FS-EE-PA microparticles were generated by two methods: In the first method, a suspension comprising 0.5 g of FS and 0.5 g of EE in 5 mL of ethanol was decanted to dry on a glass Petri dish. Upon solvent evaporation, the milky suspension became thick and viscous, eventually turning into a transparent solid solution (SS) that adhered strongly to the glass Petri dish. The SS was redissolved in acetone, and the transparent solution was added to the molten PA, and maintained at 65 °C. On contact, the acetone quickly vaporised off, and the resultant soft mass became a white brittle solid following further drying at ambient temperature on baking paper. The solid was pulverised using a mortar and pestle, and then sieved (pore diameter 355 µm) to obtain microparticles (Figure 16). The FS:EE:PA microparticles were assessed by the research team to be effective at masking the taste of FS. However, the 2-step manufacturing process was not robust, as the powders fabricated under relatively high environmental humidity had a yellow discolouration indicative of FS degradation when stored overnight.

The second method employed one-step processing by triturating FS, EE, and PA (1:1:1 *w*/*w*/*w*) together in either ethanol or acetone and allowing the suspensions to dry over 24 h in the mortar at ambient temperature. The resultant powders after sieving were assessed by the research team to have similar taste profiles to those of the powders fabricated using the 2-step manufacturing process. This suggests that the prior fabrication of the SS was not a necessary step to prepare the FS:EE:PA microparticles.

We hypothesised that the taste-masking of FS achieved in the FS:EE:PA microparticles was due to the complex interactions of FS, PA, and EE [35]. An acid–base reaction involving the carboxylic acid of palmitic acid (PA-COOH) and the tertiary amine group of EE (Equation (1)) led to the formation of PA-COO^−^, which reacted with the Na^+^ counterion of FS (F-COONa) to form the sodium palmitate soap (Equation (2)), while the protonated EE (EE-H^+^) interacted with the ionised FS (F-COO^−^) (Equation (3)).

PA-COOH + EE ⇄ PA-COO^−^ + EE-H^+^ (1)

F-COONa + PA-COO^−^- ⇄ F-COO^−^ + PA-COONa (2)

F-COO^−^ + EE-H^+^+ ⇄ [F-COO^−^-EE-H^+^] (3)

However, the issue with the single-step fabrication process was that the suspension where FS, EE, and PA were present as solid particles of different sizes did not provide optimal conditions for the molecular interactions. A ternary solution where the ingredients were molecularly dispersed was preferred over the suspension to optimise the molecular interactions. The following section explains the application of a mixture design to select the optimal solvent system to assure consistent molecular interactions among the 3 components.

#### 4.2.2. Overview of Experimental Design

An optimal solvent system may be applied to a single-phase ternary solution comprising all 3 ingredients (FS, EE, and PA), or to a two-phase system, each phase of which comprises different ratios of the components that are then mixed to yield the final ternary system. A mixture design was applied to determine the optimal solvent in terms of the proportion of ethanol to acetone to use for each binary phase, as well as the final ternary phase. For the two-phase systems, in order to include all possible combinations of the 3 components, 3 binary phases were generated, each comprising two components (FS + EE, FS + PA, and PA + EE), along with 3 single-component phases (FS, EE, and PA). The final phase, a suspension obtained by mixing the binary and single component solutions, was a ternary system where all 3 components were present, and this suspension was dried to generate the FTMs. The proportions of ethanol and acetone were independent variables and ranged in value from 0 to 1. The dependent variable (i.e., response) for the single and binary phases was the absorbance at 550 nm (visible spectrum), which served as a surrogate for opacity. Higher absorbance was indicative of greater opacity and, therefore, lower solubility of the components in the solvent system. The opacity (absorption at 550 nm) was used as a response in the mixture model to predict the optimal solvent ratio for solubilising the components in the binary and single-component solutions. The dependent variables (responses) for the FTM were drug-loading efficiency (%) and summation of AUC for all peaks measured at 343 nm (AUC_343_). The FS content in the FTMs was quantified at 225 nm using a validated HPLC assay, and the drug-loading efficiency was calculated using Equation (4), with higher drug-loading efficiency indicating a more stable ternary phase.

Drug loading efficiency (% *w*/*w*) = (Drug load measured in microparticles (mg))/(Drug amount added in microparticles (mg)) × 100 (4)

The UV spectra of FTM samples obtained via diode-array detection showed the intensity of discolouration of the samples to be correlated with the height of the absorption peak at 343 nm. Thus, the AUC_343_ was used as a surrogate to measure the intensity of yellow discolouration of the FTMs. AUC_343_ was quantified using the validated HPLC assay for FS, with eluent absorption measured at 343 nm. For the FTMs, the aim was to determine the solvent ratio that minimised the AUC_343_ and maximised the drug-loading efficiency.

#### 4.2.3. Determination of Solvent Systems to Solubilise Single and Multiple Components

##### One-Phase System (Single Ternary Phase)

The three components (0.2 g of FS, 0.16 g of EE, and 0.12 g of PA) were accurately weighed into a 200 mL beaker, and the specified solvent was added in increments of 1 mL with agitation at 250 rpm using a magnetic stirrer. The mixture was visually examined after the addition of each solvent, and the volume of solvent that turned the suspension into a solution with no visible particles was noted as the volume required for solubilisation. The experiment was aborted when solubilisation was not achieved after 25 mL of the specified solvent was added.

##### Two-Phase Systems (Combination of Binary and Single-Component Phases)

The 3 components were divided into two phases, as shown in Table 7. For each option, the components for the phases were weighed into 10 mL glass vials, and the specified solvent was added. A mixture design with 8 runs was generated using the Design-Expert 12 software (Stat-Ease Inc., Minneapolis, MN, USA). A simplex lattice design was used, where the design points were evenly distributed over the ethanol proportion ranging from 0 to 1 (Table 8). The experiment was performed as a single block with three replicate points, and all runs were randomised. Statistical analysis was performed using the Design-Expert 12 software.

A preliminary experiment was conducted to determine the minimum volume of liquid that would allow the powders to be rendered into a paste that could be triturated using a magnetic stirrer. This was performed by weighing 0.2 g of FS, 0.16 g of EE, and 0.12 g of PA into a 10 mL glass vial and adding ethanol in increments of 20 µL with magnetic stirring. It was found that the minimum volume of ethanol required to form a workable paste was 3.68 mL/g. Given that the maximum amount of powder was 0.2 g in phase 1 (Option 1) and 0.36 g in phase 2 (Option 3), the specified solvent was added at 1 mL to phase 1 and at 2 mL to phase 2 for all 3 options.

Following the addition of the solvent, the sample vials were sealed with paraffin film, stirred on a magnetic stirrer for 10 min, and then loaded at 30 µL into a 96-well microplate, auto-mixed for 50 s with a plate cover in place to prevent solvent evaporation, and measured for absorbance at 550 nm using a microplate reader (POLARstar OPTIMA, BMG Labtechnologies Pty. Ltd., Mornington, Australia). The data were analysed using the OPTIMA software (version 2.20R2, BMG LABTECH, Melbourne, Australia), with neat solvents as controls.

##### Determination of Optimal Solvent for a Ternary System to Generate Stable FTMs with Maximised Drug-Loading Efficiency

To determine the optimal solvent for a ternary system to generate stable FTMs with maximised drug-loading efficiency, ternary suspensions were investigated where the combined FS, EE, and PA powders were triturated with 3 mL of the specified solvent. Similarly, a mixture design with 8 runs was generated using the Design-Expert 12 software (Stat-Ease Inc., MN, USA). A simplex lattice design was used, where the design points were evenly distributed over the ethanol proportion ranging from 0 to 1 (Table 9). The experiment was performed as a single block with three replicate points, and all runs were randomised. Statistical analysis was performed using the Design-Expert 12 software.

##### Preparation of FTM

The 3 components were ground into fine powders using a mortar and pestle and accurately weighed (0.2 g of FS, 0.16 g of EE, and 0.12 g of PA) into a 10 mL glass vial. The specified solvent was added at 3 mL, and the vial was sealed with paraffin film. The mixture was agitated for 10 min using a magnetic stirrer to achieve a homogeneous suspension, which was then decanted onto baking paper to dry at ambient temperature for 48 h. The resultant powder was milled using a mortar and pestle, sieved (355 µm), and transferred into a 10 mL vial with a lid for storage at ambient temperature. Samples were monitored for weight until no further weight change occurred with storage time, and the final samples, abbreviated as T1–T8, were analysed.

##### High-Performance Thin-Layer Chromatography

High-performance thin-layer chromatography (HPTLC) is a type of thin-layer chromatography (TLC) with enhanced features that include automation of sample application and image analysis [36]. The automated sample application allows for greater control of the amount and position of the sample applied on the TLC plate, and multiple samples can be applied on the same plate for more robust comparative analysis. Following sample development, the UV spectra of chromatographic bands can be acquired via diode-array detection and conveniently compared using the HPTLC software (VisionCATS 3.2 SP 2, CAMAG, Muttenz, Switzerland). While the HPTLC method can be validated for quantitative analysis, for this study, HPTLC was used only for qualitative comparisons of images and UV spectra. Quantitative data were subsequently obtained with a validated HPLC method.

FTMs (T1–T8, 10 mg) were separately dissolved by sonication for 30 min with 1 mL of ethanol in HPLC vials. An FS solution (where the solvent was 0.8 mL of ethanol and 0.2 mL of acetone) served as the control sample. The mobile phase comprised 4:1 *v*/*v* methanol and dichloromethane. Samples were applied at 20 µL on an HPTLC plate (silica gel 60 F254 HPTLC plate, 20 × 10 cm) using a semi-automated application device (Linomat 5, CAMAG, Muttenz, Switzerland) and eluted in an automated development chamber (ADC2, CAMAG) preconditioned to 33% RH. The images of the applied samples were taken under white light (TLC visualiser 2, CAMAG) prior to elution, and the images were processed with ImageJ (Version 1.53a, U.S. National Institutes of Health, Bethesda, MD, USA). Following elution, the UV absorption spectra of chromatographic bands eluted at the same retention factor as the FS control sample were obtained using the diode-array detector and digitally processed (visionCATS, CAMAG). The images of T1–T8, FS, EE, and PA powders placed on another HPTLC plate were also taken under white light using the HPTLC imaging device and augmented using ImageJ.

##### Drug-Loading Efficiency and AUC_343_

FTMs (T1–T8, 15 mg) were dissolved by sonication for 30 min with 1.5 mL of ethanol, and 100 µL samples were aliquoted into 10 mL glass test tubes. Each sample was mixed by vortexing for 1 min with 4 mL of diluent (25 mL of acetonitrile and 75 mL of 20 mM potassium phosphate buffer (pH 5.0), with 0.75 g of KCl added to reduce ionic interactions among the components). The diluted samples after filtration (0.2 μm × 13 mm nylon syringe filter, Agilent Technologies Australia, Sydney, NSW, Australia) were analysed by HPLC. Standard solutions were prepared by diluting a stock solution (10 mg of FS, 8 mg of EE, and 6 mg of PA in 1.5 mL of ethanol) with the diluent to give concentrations in the range of 0.03–0.16 mg/mL.

The HPLC assay for FS was adapted from the British Pharmacopoeia (BP) method, with slight modifications [37]. HPLC analysis was performed on an integrated Agilent 1260 Infinity II binary LC system (Agilent Technologies Australia, Sydney, NSW, Australia) with an Agilent 1260 Infinity diode-array detector. Chromatographic separation was carried out at 20 °C on a reversed-phase C18 column (Hypersil ODS column, 150 × 4.5 mm, 5 µm particle size, Thermo Fisher Scientific, WA, Australia) coupled with a Hypersil Classical C18 Guard Cartridge (5 µm, 10 × 4.6 mm). Isocratic elution was applied with a mobile phase comprising 25:75 *v*/*v* acetonitrile and potassium phosphate buffer (20 mM, pH 5.0), at a flow rate of 1 mL/min. The sample injection volume was 20 µL at ambient temperature, and the eluent was monitored at 225 nm for FS quantification and at 343 nm for determination of the AUC_343_. The total run time was 20 min. Chromatographic data were collected and processed using the Agilent OpenLAB CDS software (version 2.4, Melbourne, Australia).

#### 4.2.4. Confirmation Study of FTMs Produced by the Optimised Fabrication Process

##### Preparation of FTMs

Three batches of FTMs were prepared on the same day (intraday), and another three batches were prepared on a separate day (interday), using the optimal solvent system. Briefly, an FS solution (0.2 g in 1 mL of 1:4 *v*/*v* ethanol:acetone) and a binary PA-EE solution (0.16 g of EE and 0.12 g of PA in 2.2 mL of ethanol) were separately prepared in 10 mL vials. The FS solution was added to the binary solution under magnetic agitation for 5 min at 350 rpm, and the suspension was decanted onto baking paper to dry overnight at ambient temperature. The resultant particles were milled in a mortar, and after sieving (355 µm) they were transferred into 10 mL vials with lids for storage at ambient temperature. The sample weight was monitored until no further weight change occurred before the samples were further analysed.

##### Drug-Loading Efficiency

The drug-loading efficiency for each batch of FTMs was measured using the validated HPLC method.

##### UV Spectra

FTM (15 mg) and FS (5 mg) samples were separately dissolved by sonication for 30 min in 1.5 mL of ethanol in HPLC vials. The UV spectra of the solutions after 1:50 dilution with ethanol were acquired at 200 nm to 400 nm (VARIAN Carry 50 Bio, Agilent, Melbourne, Australia), and analysed (Cary WinUV software, version 5.0.0.999, Melbourne, Australia).

##### DSC

A ternary physical mixture (PM) was prepared by triturating FS, EE, and PA at a weight ratio of 1:0.8:0.6 for 3 min using a mortar and pestle [38]. Binary PMs of FS-EE (1:0.8 *w*/*w*), EE-PA (0.8:0.6 *w*/*w*), and FS-PA (1:0.6 *w*/*w*) were prepared similarly. The FTM and PM samples were weighed (2–5 mg) and sealed into aluminium pans (40 µL). DSC analysis was conducted under nitrogen gas purging (50 mL/min) and at heating rates of 1–20 °C/min over the temperature range of −10 °C to 170 °C (Discovery DSC 25, TA instruments, Delaware, USA, equipped with liquid nitrogen quench -cooling). An empty pan served as a reference. The thermograms were analysed using Trios software (version 5.0.0., DE, USA).

## 5. Conclusions

A promising taste-masked powder comprising microparticles of FS, EE, and PA was successfully prepared that did not require a solid solution of FS and EE to be fabricated as an initial step. The fabrication process was successfully optimised using the mixture design methodology to determine optimal solvent ratios to solubilise the components, as well as the optimal solvent ratios to prepare a stable FTM product with maximised drug-loading efficiency and minimised drug degradation. This fabrication process involves dissolving FS in 20:80 *v*/*v* ethanol:acetone, co-dissolving EE and PA in ethanol, and mixing the two solutions to give a ternary mixture of 75:25 *v*/*v* ethanol:acetone. Drying of the ternary mixture, followed by grinding and sieving (355 µm), provided a powder that appeared to effectively mask the bitter taste of FS. The optimised fabrication process is robust, reproducible, and potentially scalable.

## Figures and Tables

**Table 1:** Opacity (UV absorbance at 550 nm) obtained for phase 1 and phase 2 for Option 1 of the two-phase systems when mixed with specified solvents. Phase 1 was mixed with 1 mL of solvent, while phase 2 was mixed with 2 mL of solvent. FS = flucloxacillin sodium, EE = Eudragit EPO, PA = palmitic acid.

Design Point	Proportion of Ethanol	Proportion of Acetone	Opacity for Phase 1 (FS)	Opacity for Phase 2 (EE + PA)
1	1	0	2.96	0.06
2	0.5	0.5	2.43	0.03
3	0	1	2.32	0.55
4	0.5	0.5	2.28	0.05
5	0.75	0.25	2.86	0.03
6	1	0	2.41	0.05
7	0.25	0.75	0.14	0.41
8	0	1	2.68	0.46

**Table 2:** Coefficients and confidence intervals (CIs) for the mixture model for phase 1 and 2, where the independent variables are the proportions of solvents and the respondents are opacity for phase 1 and phase 2 measured as visible spectral absorption at 550 nm on a microplate reader. A: proportion of ethanol; B: proportion of acetone volume.

*p*-Value for Model for Phase 1: 0.02		
Model Terms for Phase 1	Coefficient Estimate	95% CI
A	2.69	2.07–3.30
B	2.50	1.88–3.12
AB	−0.94	−3.97–2.09
AB(A-B)	13.99	6.99–20.99
AB(A-B)^2^	−19.52	−36.18–−2.86
Model Terms for Phase 2		
***p*** **-Value for Model for Phase 2: 0.025**		
A	0.05	−0.094–0.19
B	0.53	0.39–0.68
AB	−0.77	−1.43–−0.11

**Table 3:** AUC_343_ obtained for powders obtained by drying (at ambient temperature for 48 h) ternary suspensions prepared by triturating 0.2 g of FS, 0.16 g of EE, and 0.12 g of PA with 3 mL of the specified solvent. AUC_343_ is the UV absorption of all peaks measured at 343 nm using the HPLC assay developed for flucloxacillin sodium. FS = flucloxacillin sodium, EE = Eudragit EPO, PA = palmitic acid.

Sample Number	Proportion of Ethanol	Proportion of Acetone	AUC_343_ of Ternary Phase
T1	1	0	0.86
T2	0.5	0.5	2.27
T3	0	1	0.45
T4	0.5	0.5	2.22
T5	0.75	0.25	0.37
T6	1	0	0.39
T7	0.25	0.75	0.98
T8	0	1	1.65

**Table 4:** Coefficients and confidence intervals (CIs) for the mixture model, where the independent variables are the proportions of solvents and the respondents are the FS loading efficiency (%) and AUC_343_ for the ternary samples of flucloxacillin sodium (FS), Eudragit EPO, and palmitic acid (T1–T8). AUC_343_ is the UV absorption of all peaks measured at 343 nm using the HPLC assay developed for FS. A: proportion of ethanol; B: proportion of acetone volume.

*p*-Value for Model of % FS Loading Efficiency: <0.0359; *p*-Value for Lack of Fit: 0.1976		
Model Terms of % FS Loading Efficiency	Coefficient Estimate	95% CI
A	35.84	33.61–38.07
B	33.38	31.15–35.61
AB	12.52	2.19–22.85
***p*** **-Value for Model of AUC_343_: <0.0001; *p*-Value for Lack of Fit: 0.4461**		
**Model Terms of AUC_343_**	**Coefficient Estimate**	**95% CI**
A	3.82	−2.86–4.78
B	22.47	21.50–23.43
AB	−15.29	−19.63–−10.95
AB(A-B)	−14.31	−25.27–−3.35

**Table 5:** Volumes of ethanol and acetone required to fulfil the optimal solvents predicted to solubilise phase 1 and phase 2, and to produce stable FTMs using 1 g of FS, 0.8 g of EE, and 0.6 g of PA as starting materials. FS = flucloxacillin sodium, EE = Eudragit EPO, PA = palmitic acid.

Phase	Ethanol (mL)	Acetone (mL)	Ethanol:Acetone (*v*/*v*)	Weight of Component(s) (g)	Total Solvent (mL) (Equivalent Weight (g))
FS (Phase 1)	0.74	2.94	0.2:0.8	1	3.68 (2.88)
EE + PA (Phase 2)	8.10	0	1:0	1.4	8.1 (6.40)
Final Ternary Mixture	8.83	2.94	0.75:0.25	2.4	11.8 (9.27)

**Table 6:** Drug-loading efficiency in 3 independent batches of FTMs produced on the same day and over 2 different days. Triplicate samples were withdrawn from each batch of FTMs to determine the drug-loading efficiency via a validated HPLC assay.

	Flucloxacillin Sodium Loading Efficiency in FTMs (% *w*/*w*) (*n* = 3, Mean ± SD)	
	Day 1	Day 2
Batch 1	39.6 ± 1.0	39.9 ± 0.5
Batch 2	40.8 ± 1.0	39.3± 0.8
Batch 3	39.6 ± 1.0	39.1 ± 0.9

**Table 7:** Components present in phase 1 (single component) and phase 2 (two components) employed for the two-phase experiments.

Option Number	Component in Phase 1 (Mass in g)	Components in Phase 2 (Mass in g)
1	FS (0.2)	EE (0.16) + PA (0.12)
2	EE (0.16)	FS (0.2) + PA (0.12)
3	PA (0.12)	FS (0.2) + EE (0.16)

**Table 8:** Simplex lattice mixture design with 8 experimental runs involving different proportions (*v*/*v*) of ethanol and acetone to determine optimal solvent systems to solubilise the components of each phase.

Sample Number	Proportion of Ethanol	Proportion of Acetone
1	1	0
2	0.5	0.5
3	0	1
4	0.5	0.5
5	0.75	0.25
6	1	0
7	0.25	0.75
8	0	1

**Table 9:** Simplex lattice mixture design with 8 experimental runs involving different proportions of ethanol and acetone to determine the optimal solvent for a ternary system to generate stable FTMs.

Sample Number	Proportion of Ethanol	Proportion of Acetone
T1	1	0
T2	0.5	0.5
T3	0	1
T4	0.5	0.5
T5	0.75	0.25
T6	1	0
T7	0.25	0.75
T8	0	1

## Data Availability

Data is contained within the article.
